# Difference of Progression to AIDS According to CD4 Cell Count, Plasma HIV RNA Level and the Use of Antiretroviral Therapy among HIV Patients Infected through Blood Products in Japan

**DOI:** 10.2188/jea.16.101

**Published:** 2006-05-19

**Authors:** Miyuki Kawado, Shuji Hashimoto, Takuhiro Yamaguchi, Shin-ichi Oka, Kazuyuki Yoshizaki, Satoshi Kimura, Katsuyuki Fukutake, Satoshi Higasa, Takuma Shirasaka

**Affiliations:** 1 Department of Hygiene, Fujita Health University School of Medicine.; 2 Department of Biostatistics / Epidemiology and Preventive Health Sciences, School of Health Science and Nursing, University of Tokyo.; 3 AIDS Clinical Center, International Medical Center of Japan.; 4 Department of Medical Science I, School of Health and Sport Sciences, Osaka University.; 5 Department of Laboratory Medicine, Tokyo Medical University Hospital.; 6 Department of Internal Medicine, Hyogo College of Medicine.; 7 Osaka National Hospital.

**Keywords:** HIV, Acquired Immunodeficiency Syndrome, Blood Coagulation Factors, CD4 Lymphocyte Count, Antiretroviral Therapy, Highly Active

## Abstract

**BACKGROUND:**

It is important to examine progression to acquired immunodeficiency syndrome (AIDS) or death and its predictors among human immunodeficiency virus (HIV) infected persons before and after the introduction of the highly active antiretroviral therapy (HAART) available in Japan since 1997.

**METHODS:**

The data used were from a survey of persons with HIV infected through blood coagulation factor products in Japan. Progression to AIDS or death during two periods, between January 1994 and March 1997, and between April 1997 and March 2002, were observed.

**RESULTS:**

The AIDS-free proportion after 3 years was 74% among 417 participants for the earlier period and 94% among 605 participants in the later one. The hazard ratio of low CD4 cell count (less than 200 cells/*μ*L) was 50.8 for the earlier period and 4.7 for the later one compared with that of 500 cells/*μ*L or more. After adjustment by plasma HIV RNA levels and use of antiretroviral therapy, the hazard ratios of the low CD4 cell count for the later period were still significant.

**CONCLUSION:**

The AIDS-free proportion among people with HIV infected through blood products in Japan largely increased after the introduction of HAART. The CD4 cell count remains an important predictor of future progression, but its importance might be less because of HAART.

It is a great challenge for both the public health and medical fields to discover the potential human immunodeficiency virus (HIV) infections^1^ and to prevent HIV-infected persons from progressing to acquired immunodeficiency syndrome (AIDS) or death. In recent years, highly active antiretroviral therapy (HAART) including combination regimens such as two nucleoside reverse transcriptase inhibitors plus one protease inhibitor have become available.^2^ It is well-known that they have had a significant impact on preventing or delaying AIDS progression for individuals with HIV,^3^^-^^5^ whereas their actual impact on the entire HIV-infected population has not been sufficiently evaluated except several European countries and the United States. Also in Japan, there is no precedent in terms of observational study.

The CD4 cell count and plasma HIV-RNA level were important for predicting future progression to AIDS or death among HIV-infected persons before the introduction of HAART,^6^^-^^8^ but their implications may have been reduced since then.^9^^-^^11^ There are a few reports on the predictors of AIDS progression before and after HAART became widely available.^9^^,^^11^

In the present study, we examined the progression to AIDS or death before and after the widespread use of HAART among people with HIV infected through blood products in Japan. The effect of the CD4 cell count as a predictor of progression to AIDS or death before and after HAART usage was evaluated. The effects of plasma HIV RNA level and use of antiretroviral therapy after HAART became available were also examined.

## METHODS

### Survey and Research Program for People with HIV Infected through Blood Products in Japan

In Japan, a survey and research program for people with HIV infection through the use of contaminated blood coagulation factor products has been carried out since 1993 fiscal year with the support of the Ministry of Health and Welfare.^12^^-^^14^ This program is intended to help prevent them from developing HIV-infected symptoms in daily living by providing health management expenses. For this research, subjects were requested to submit reports filled out by their treatment physician on a quarterly basis, which included CD4 cell count and administered antiretroviral drugs. The plasma HIV RNA level was appended to this report from the second quarter of 1997. If subjects were diagnosed with AIDS,^15^ they were excluded from this survey. The date of the diagnosis of AIDS or death was ascertained in the survey. Details of the survey had been described elsewhere.^12^^-^^14^

### Data Analysis

The data from the survey mentioned above were made available, including sex, age, CD4 cell count, plasma HIV RNA level, antiretroviral therapy status, and the date of the diagnosis of AIDS onset or death. No personal identifiers such as name or address were included. We drew two subset cohorts from that data for our analysis. One cohort consisted of participants on January 1, 1994. The other consisted of participants as of April 1, 1997 because HAART became available in Japan in 1997. The first subset cohort was used in the analysis of progression to AIDS or death before 1997, and the second subset cohort was used in that after 1997.

For the analysis of progression to AIDS or death before 1997, the data of progression to AIDS or death between January 1, 1994 and March 31, 1997 were used. AIDS-free proportion by CD4 cell count in the first quarter of 1994 was estimated using Kaplan-Meier methods. The Cox proportional hazards model was used to estimate the hazard ratio of the CD4 cell count for progression to AIDS or death and its 95% confidence interval. Because the data did not include plasma HIV RNA levels, and use of antiretroviral therapy was rare in the first quarter of 1994, we did not use these variables in this analysis.

For the analysis of progression to AIDS or death after 1997, the data between April 1, 1997 and March 31, 2002 were used. AIDS-free proportions, hazard ratios for progression to AIDS or death and their 95% confidence intervals were estimated by CD4 cell count, plasma HIV RNA level and the use of antiretroviral therapy in the second quarter of 1997. Hazard ratios by combinations of these three variables were also estimated.

The CD4 cell count was divided into four categories: less than 200, 200-349, 350-499, and 500 cells/*μ*L or more. The plasma HIV RNA level was divided into six categories: less than 400, 400-999, 1,000-4,999, 5,000-9,999, 10,000-49,999 and 50,000 copies/mL or more. Use of antiretroviral therapy was classified into five categories: given no treatment (No treatment), treatments including only one nucleoside reverse transcriptase inhibitor (1 NRTI), those including only two NRTIs (2 NRTIs), those including at least two NRTIs and one protease inhibitor (2 NRTIs + 1 PI), and other treatments (Other treatments). For estimating hazard ratios by combinations of the three variables, we divided each variable into two categories before putting them together; CD4 cell count (less than 200 vs. 200 cells/*μ*L or more), plasma HIV RNA level (less than 50,000 vs. 50,000 copies/mL or more), and the use of antiretroviral therapy (2 NRTIs + 1 PI vs. not 2 NRTIs + 1 PI).

All analyses were conducted using SAS^®^ software, version 8.2 (SAS Institute, Inc., Cary, NC, USA).^16^

## RESULTS

### Analysis of Progression to AIDS or Death before 1997

The number of participants as of January 1, 1994 was 417 (415 males and 2 females). Mean age was 27.9 (standard deviation = 10.7) years. Of these, 113 participants progressed to AIDS or death, and no participant was lost to follow-up by March 31, 1997. The proportion of AIDS-free participants after 3 years was 0.74.

**Figure 1.**
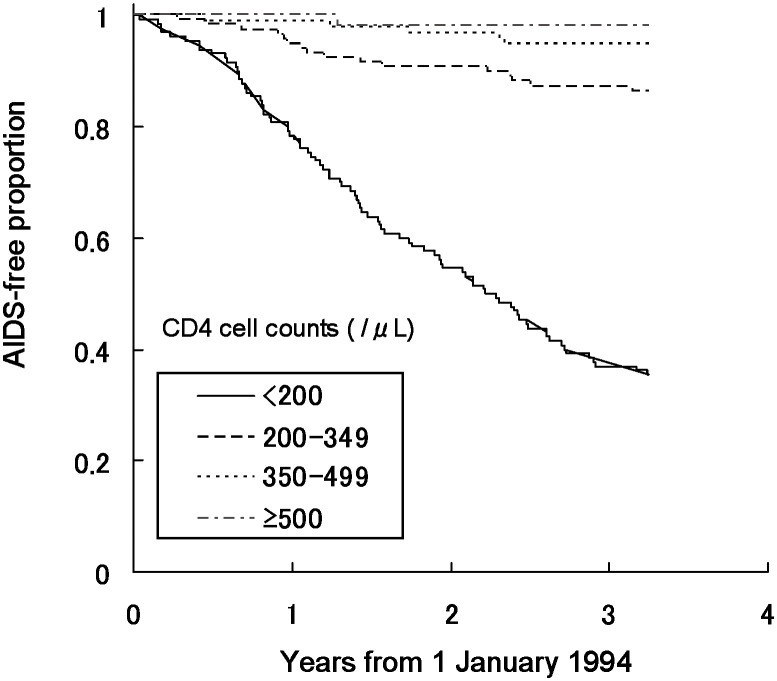
AIDS-free proportion between January 1, 1994 and March 31, 1997 by CD4 cell count in the first quarter of 1994.

Figure 1 shows AIDS-free proportions before April 1997 by CD4 cell counts in the first quarter of 1994. Among 400 participants whose CD4 cell count was available, the AIDS-free proportion decreased rapidly each year where the CD4 cell count was less than 200 cells/*μ*L.

As shown in Table 1, the hazard ratio of progression to AIDS or death before April 1997 was 50.8 (p<0.01) in CD4 cell counts of less than 200 cells/*μ*L and 7.7 (p=0.05) in those of 200-349 cells/*μ*L compared with those of 500 cells/*μ*L or more.

**Table 1.** Hazard ratios of CD4 cell count in the first quarter of 1994 for progression to AIDS or death before 1997.

CD4 cell count (cells / *μ*L)	n	Hazard ratio	95% confidence interval	p-value
<200	125	50.79	7.06 - 365.30	<0.01
200 – 349	118	7.73	1.03 - 58.25	0.05
350 – 499	96	2.81	0.33 - 24.01	0.35
≥500	53	1	(reference)	

### Analysis of Progression to AIDS or Death after 1997

The number of participants at April 1, 1997 was 605 (583 males and 22 females). Mean age was 29.7 (standard deviation = 10.1) years. Of these, 65 participants progressed to AIDS or death and 1 participant was lost to follow-up by March 31, 2002. The proportion of AIDS-free participants was 0.94 after 3 years and 0.89 after 5 years.

**Figure 2.**
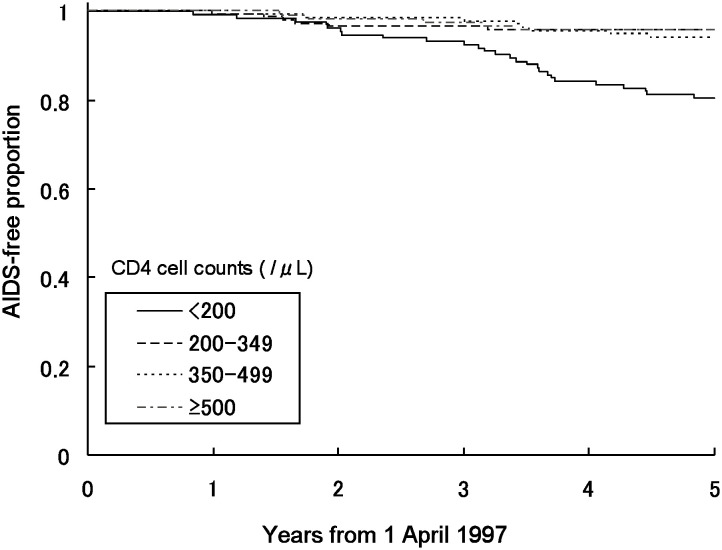
AIDS-free proportion between April 1, 1997 and March 31, 2002 by CD4 cell count in the second quarter of 1997.

**Figure 3.**
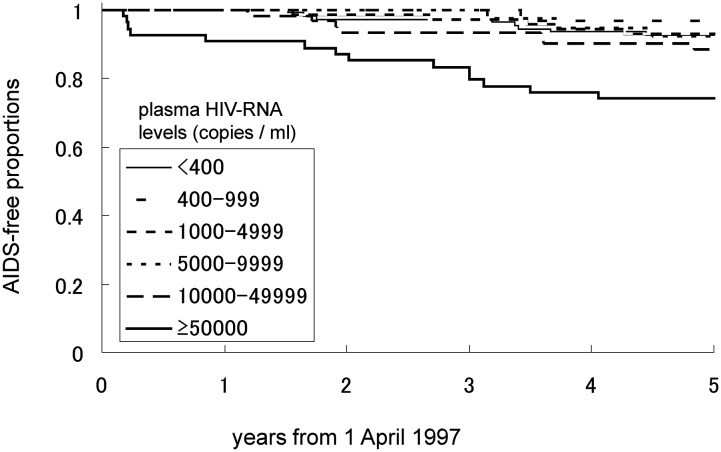
AIDS-free proportion between April 1, 1997 and March 31, 2002 by plasma HIV RNA level in the second quarter of 1997.

**Figure 4.**
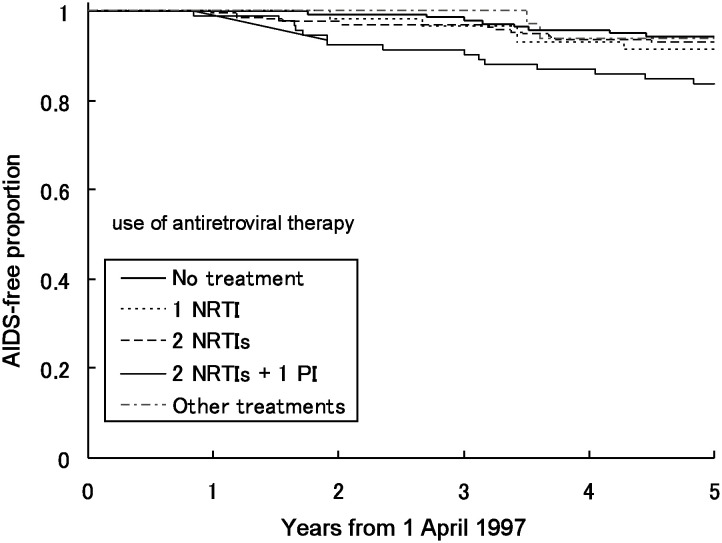
AIDS-free proportion between April 1, 1997 and March 31, 2002 by the use of antiretroviral therapy in the second quarter of 1997. * 1 NRTI: treatments including only one nucleoside reverse transcriptase inhibitor. 2 NRTIs: treatments including only two nucleoside reverse transcriptase inhibitors. 2 NRTIs + 1 PI: treatments including at least two nucleoside reverse transcriptase inhibitors and one protease inhibitor.

Figures 2, 3 and 4 show the AIDS-free proportion between April 1997 and March 2002 by CD4 cell count, plasma HIV RNA level, and the use of antiretroviral therapy at the second quarter of 1997, respectively. The number of participants in this analysis was 525 for CD4 cell count, 357 for plasma HIV RNA level and 542 for use of antiretroviral therapy because of missing data. The AIDS-free proportion was lower where the CD4 cell count was less than 200 cells/*μ*L, with plasma HIV RNA levels of 50,000 copies/mL or more and also lower in 2 NRTIs + 1 PI than in the others.

Table 2 shows the hazard ratios of CD4 cell count, plasma HIV RNA level and use of antiretroviral therapy at the second quarter of 1997 for progression to AIDS or death between April 1997 and March 2002. The hazard ratio was 4.7 (p<0.01) in a CD4 cell count of less than 200 cells/*μ*L compared with those of 500 cells/*μ*L or more, 2.9 (p=0.02) in those of 50,000 copies/mL or more compared with those with less than 400 copies/mL, and 3.1 (p=0.01) in 2 NRTIs + 1 PI compared with no treatment.

**Table 2.** Hazard ratios of CD4 cell count, plasma HIV RNA level and the use of antiretroviral therapy in the second quarter of 1997 for progression to AIDS or death after 1997.

variable	n	Hazard ratio	95% confidence interval	p-value
CD4 cell count (cells / *μ*L)				
<200	133	4.74	1.82 - 12.35	<0.01
200 – 349	144	0.96	0.29 - 3.13	0.94
350 – 499	134	1.36	0.45 - 4.16	0.59
≥500	114	1	(reference)	

Plasma HIV RNA level (copies / mL)				
<400	108	1	(reference)	
400 – 999	30	0.44	0.06 - 3.50	0.44
1,000 – 4,999	71	0.94	0.31 - 2.88	0.92
5,000 – 9,999	38	1.04	0.28 - 3.93	0.95
10,000 – 49,999	60	1.61	0.58 - 4.45	0.36
≥50,000	50	2.94	1.16 - 7.45	0.02

Use of antiretoroviral therapy*				
No treatment	144	1	(reference)	
1 NRTI	58	1.57	0.52 - 4.81	0.43
2 NRTIs	214	1.28	0.54 - 3.03	0.57
2 NRTIs + 1 PI	93	3.13	1.33 - 7.37	0.01
Other treatments	33	1.08	0.23 - 5.10	0.92

*1 NRTI: treatments including only one nucleoside reverse transcriptase inhibitor.

2 NRTIs: treatments including only two nucleoside reverse transcriptase inhibitors.

2 NRTIs + 1 PI: treatments including at least two nucleoside reverse transcriptase inhibitors and one protease inhibitor.

Table 3 shows the hazard ratios of the combinations of CD4 cell count, plasma HIV RNA level, and use of antiretroviral therapy in the second quarter of 1997 for progression to AIDS or death between April 1997 and March 2002. The hazard ratio was significantly higher in each of the combinations with a CD4 cell count of less than 200 cells/*μ*L than in the combination with CD4 cell counts of 200 cells/*μ*L or more, plasma HIV RNA levels of less than 50,000 copies/mL, and not 2 NRTIs + 1 PI.

**Table 3.** Hazard ratios of combination of CD4 cell count, plasma HIV RNA level and the use of antiretroviral therapy in the second quarter of 1997 for progression to AIDS or death after 1997.

CD4 cell counts (cells / *μ*L)	Plasma HIV RNA level (copies / mL)	Use of antiretroviral treatment*	n	Hazard ratio	95% confidence interval	p-value
≥200	<50,000	not 2NRTIs + 1 PI	216	1	(reference)	
≥200	<50,000	2 NRTIs + 1 PI	27	2.35	0.65 - 8.41	0.19
≥200	≥50,000	not 2NRTIs + 1 PI	19	1.04	0.13 - 8.01	0.97
≥200	≥50,000	2 NRTIs + 1 PI	4	0.00	0.00 - －	0.99
<200	<50,000	not 2NRTIs + 1 PI	41	2.89	1.07 - 7.81	0.04
<200	<50,000	2 NRTIs + 1 PI	23	3.51	1.12 - 11.02	0.03
<200	≥50,000	not 2NRTIs + 1 PI	13	5.02	1.40 - 18.01	0.01
<200	≥50,000	2 NRTIs + 1 PI	13	12.42	4.59 - 33.65	<0.01

* 2 NRTIs + 1 PI: treatments including at least two nucleoside reverse transcriptase inhibitors and one protease inhibitor.

## DISCUSSION

Among people with HIV infected through blood coagulation factor products, the AIDS-free proportion before 1997 was markedly lower than that after 1997, when HAART was widely used in Japan. This would reflect the strong effect of HAART for preventing or delaying the progression to AIDS or death. Several studies conducted in Europe or the United States indicated results similar to those of the present study.^9^^,^^11^

The hazard ratio for progression to AIDS or death before 1997 was 50.8 in CD4 cell counts of less than 200 cells/*μ*L compared with those of 500 cells/*μ*L or more. This result was consistent with the previous reports,^9^^,^^11^ showing that a low CD4 cell count was an important predictor of future progression to AIDS or death among HIV-infected persons before the introduction of HAART. The hazard ratio of a CD4 cell count of less than 200 cells/*μ*L for progression to AIDS or death after 1997 was 4.7. These results suggested that while the CD4 cell count was still an important predictor of future progression to AIDS or death after the introduction of HAART, its importance has lessened because of the introduction of HAART.^17^

The AIDS-free proportion after 1997 was lower in plasma HIV RNA levels of 50,000 copies/mL or more than in other levels. The hazard ratio of the combination of higher plasma HIV RNA level and lower CD4 cell count was significant, whereas that of the combination of higher plasma HIV RNA level and higher CD4 cell count was not. Thus, the plasma HIV RNA level, as a predictor of future progression to AIDS or death, might be less important than the CD4 cell count. This suggestion would be consistent with the recommendations for treatments of HIV-infected persons: the CD4 cell count could be used as a marker for beginning HAART, and the plasma HIV RNA level may be used as a marker for monitoring the effect of HAART treatments.^18^^,^^19^

We observed that the AIDS-free proportion after 1997 was lower in 2 NRTIs + 1 PI than in the others. The hazard ratio of the combination of 2 NRTI + 1 PI and lower CD4 cell count was significant, but that of the combination of 2 NRTIs + 1 PI and higher CD4 cell count was not. These results do not indicate the ineffectiveness of the treatment with 2 NRTIs + 1 PI; rather, it would reflect that patients treated by 2 NRTIs + 1 PI had a higher risk of progression to AIDS or death if their CD4 cell counts after the use of HAART were still low.^5^

The present analysis has several problems and limitations. We used data from a survey of patients with HIV infection through blood coagulation factor products in Japan. All people infected with HIV through blood coagulation factor products were not subjects of this survey because they needed to make an application for participation and then had to be approved as subjects. The coverage of the subjects in April 1997 was about 80%. We extracted two subset cohorts from the data for our analysis. Therefore, the later cohort contains the former cohort’s subject who survived and did not develop AIDS until March 31, 1997. The results from one cohort might not be strictly comparable with those from the other cohort. The data on the progression to AIDS or death were ascertained in the survey, and were used as evidence for disease progression; however, some of the deaths could have been caused by other diseases without having progressed to AIDS. The data we used did not include the cause of death. In our analysis of the progression to AIDS or death, other variables such as sex and age were not included. The date of the diagnosis of HIV infection was also not included because data were not available. We focused on the effects of variables as a predictor for future progression to AIDS or death. Future progression to AIDS or death could be strongly influenced by future treatments. Most of our subjects would have received adequate treatments during the period when the progression to AIDS or death was observed.

Although our findings might be restricted by some of the problems and conditions mentioned above, this study should help to evaluate the actual impact of the widespread use of HAART on the HIV-infected population in non-Western countries, and to provide information on the importance of CD4 cell counts and plasma HIV RNA levels as predictors of future progression to AIDS or death.
